# Volumetric-Modulated Arc Radiotherapy with Daily Image-Guidance Carries Better Toxicity Profile for Higher Risk Prostate Cancer

**DOI:** 10.31557/APJCP.2021.22.1.61

**Published:** 2021-01

**Authors:** Ahmed I Ghanem, Amr A Elsaid, Mohamed A Elshaikh, Gehan A Khedr

**Affiliations:** 1 *Department of Radiation Oncology, Henry Ford Cancer Institute, Detroit, Michigan, USA. *; 2 *Department of Clinical Oncology, Faculty of Medicine, Alexandria University, Egypt. *

**Keywords:** Acute and late toxicity, image-guided radiotherapy, intermediate and high-risk

## Abstract

**Purpose:**

To compare radiotherapy-induced toxicity for localized prostate-cancer (PCa) treated with versus without daily image-guidance.

**Patients and Methods:**

We identified consecutive intermediate and high-risk localized PCa patients treated with definitive radiotherapy using intensity-modulated radiotherapy (IMRT) with variable duration of androgen-deprivation therapy (ADT) within 2015-2016 (Arm-A) and 2005-2007 (Arm-B). Arm-A cases received daily online imaging guidance (IGRT) using cone-beam computed tomography (CBCT) unlike Arm-B candidates with no daily IGRT. After reporting demographic, clinico-pathological features and treatment details, we compared acute (within 3 months post-therapy) and late RT-induced toxicities between study groups graded by RTOG/CTCAE criteria. Uni/multivariate analyses (UVA/MVA) were performed to identify independent predictors for RT-related side-effects.

**Results:**

We were able to identify 257 cases who met our inclusion criteria. Overall, median age was 73 years (48-85), 67% had intermediate-risk and 47% received ADT. Arm-A included 72 patients who received IMRT delivered using volumetric-modulated arc therapy (VMAT), whereas, Arm-B was formed of 185 cases who utilized step-and-shoot static IMRT. Clinico-pathological features and treatment details were non-different across study arms except that Arm-A had more Grade Group 3, higher median total dose (79.2 vs. 74 Gy) and more pelvic lymph-nodes RT (P<0.05). Although acute toxicity was similar across groups, Arm-B encountered higher late toxicity score, more intense late genitourinary side-effects (P=0.008), with non-different late lower-gastrointestinal toxicities. On MVA, lack of daily CBCT, African-American race and higher comorbidities were independently predictive for late toxicities.

**Conclusion:**

IMRT with daily CBCT permitted safe delivery of dose-escalated IMRT with improved toxicity profile for higher-risk prostate cancer.

## Introduction

Prostate cancer (PCa) is the most common male malignancy and the second cause of cancer death in the United States (US) with expected 248,530 new cases and 34,130 deaths in 2021 (Siegel et al., 2021). Following the 2012 update of the US Preventive Task Force against routine prostate specific antigen (PSA) screening (Moyer, 2012), there has been an increase in the proportion of higher risk localized cases especially in those >75 years at diagnosis (Hu et al., 2016).

According to the National Cancer Comprehensive Network (NCCN) guidelines the majority of PCa patients of intermediate and high-risk with long life expectancy will end up receiving either radical prostatectomy (RP), or definitive radiotherapy (RT) with or without androgen deprivation therapy (ADT) (National Comprehensive Cancer Network, 2019). Men with higher risk PCa receiving definitive RT tend to be relatively older with increased comorbidity burden compared to RP candidates, as reported in multiple population-based studies (Buglione et al., 2019; Daskivich et al., 2013).

Definitive RT modalities, techniques and doses represent an evolving field for advances and updates aiming at delivering a curative dose to the prostate with the least possible side-effects that result from irradiating nearby organs at risk (OAR) (Zaorsky et al., 2013; Zaorsky et al., 2017). Intensity modulated radiotherapy (IMRT) has been established as a standard of care for external beam RT for PCa based upon various studies that have depicted a lower rate of acute and late RT-induced toxicities compared to 3D-conformal RT (3D-CRT). Improved toxicity profile was more pronounced for the lower gastrointestinal (GI) more than genitourinary (GU) side-effects (Zelefsky et al., 2008; Michalski et al., 2013; Wortel et al, 2016). Nonetheless, none of these were randomized to compare both techniques. Nowadays, delivery of IMRT is achieved more commonly by either multiple static fields (step-and-shoot) or the evolving Volumetric Modulated Arc Therapy (VMAT) with rotating arcs (Zaorsky et al., 2017).

With the higher level of dose conformality achieved with modern RT modalities, higher doses had been tested for higher risk PCa. In fact, randomized trials comparing escalated doses (76-80 Gy) vs. lower doses (68-70 Gy) showed consistent significant improvement in biochemical control with some showing less clinical recurrences as well (Kuban et al, 2007; Heemsbergen et al, 2014). However, most patients of these studies received 3D-CRT and suffered from more side-effects in the high dose arms; particularly GI toxicities. The Radiation-Therapy Oncology Group (RTOG)-0126 trial was the only randomized trial that allowed IMRT utilization after the protocol amendment albeit 3D-CRT was received by the majority (66.8%) (Michalski et al., 2013).

With the establishment of dose-escalation and the significantly improved dosimetry and toxicity profile with IMRT, many tools have been tested to account for setup errors and inter-fractional displacement of the prostate to avoid coverage misses which may render IMRT not beneficial or even harmful. This contributed to the development of image-guided RT (IGRT) concept which involves imaging to verify the position of the prostate and surrounding OAR with the possibility of shifts to ensure proper dose delivery. Also, IGRT aids minimizing planning target volume (PTV) margins, which also improves OAR dosimetry boosting the therapeutic ratio (Zarosky et al., 2017). IGRT can be applied using radio-opaque fiducials that are aligned to PTV either using 2-D kV imaging, electronic portal imaging device (EPID), or more recently using cone-beam CT (CBCT) mounted in the radiotherapy delivery machine. CBCT can be used based on soft tissue visualization avoiding the invasive procedure for fiducials insertion (Mayyas et al., 2013; Hammoud et al., 2008). Besides, 3D-ultrasound (3D-US) can be used for IGRT in addition to the electromagnetic transponders which can provide real-time prostate position data (Scarbrough et al., 2006; Willoughby et al., 2006).

Therefore, the aim of this study is to examine the impact of contemporary IMRT with daily IGRT on acute and late treatment-related side-effects for intermediate and high-risk PCa patients treated definitively with dose-escalated RT with or without ADT, compared to a retrospective arm treated without daily IGRT in a single institution setting.

## Materials and Methods

*Patients and methods*

After obtaining institutional review board approval, we identified consecutive patients with NCCN defined intermediate and high-risk localized PCa patients treated with definitive RT delivered by IMRT with daily IGRT using CBCT between 1/2015-1/2016; Arm-A (National Comprehensive Cancer Network, 2019). As a control we queried our PCa database for a similar group of patients (Arm-B) who received IMRT without daily IGRT within 2005-2007 at the same institution. We selected this time frame as it was the latest interval before the establishment of daily CBCT as a standard of care. Arm-B cases were positioned daily relying solely on skin tattoos with inconsistent weekly/biweekly image verification for setup using on-table 3D-US or two-dimensional kV portal imaging. 

Treatment in both groups was prescribed to the entire prostate and the seminal vesicles +/- pelvic lymph-nodes (LN) according to the calculated risk and provider’s discretion and, was delivered with conventional fractionation (1.8-2.0 Gy/fraction). All patients were simulated and treated with comfortably full bladder and empty rectum without using a routine enema, and also without fiducial markers insertion. PTV was formed of the prostate and proximal seminal vesicles (CTV) in addition to 1 cm all around except posteriorly (5mm). IMRT plans were optimized to ensure adequate coverage for PTV keeping organs at risk within the predetermined constraints as possible. Dose-constraints for the rectum and urinary bladder are based on percentage of volume (V) receiving 50, 65, 70 or 75 Gy according to QUANTEC (ex: V50 and V70 for rectum and V-65 and V-70 for bladder) (Bentzen et al., 2010). ADT using gonadotrophic-releasing hormone agonist/antagonist ± initial antiandrogen phase was administered within the process of shared-decision between the patient and consultant taking into consideration risk factors, baseline comorbidities and patient preferences.

Acute and late RT-related toxicities were graded by radiation oncologists based on RTOG and Common Terminology Criteria for Adverse-Events version 4 (2010) (RTOG/CTCAE) considering the worst grade observed along follow up time focusing mainly on lower GI, GU toxicities and erectile dysfunction (Cox et al., 1995). Acute toxicity was prospectively recorded during weekly visits throughout RT course using mainly a comprehensive checklist in addition to phone calls and any post-RT visits up to 3 months. On the other hand, late side-effects were tracked >3 months post-RT through radiation-oncology and urology visits until last follow-up per patients’ electronic records. 

After reporting demographics, baseline comorbidities using Charlosn Comorbidity Index (CCI), prognostic factors and treatment details, we compared the distribution of RT toxicities between study groups (Charlson et al., 1987). In addition to individual toxicities we reported the occurrence of any grade-2 or more (G-2+) side-effects, we calculated a total toxicity score including maximal grade of common toxicities per patient. 

We used Chi-Squared or Fisher-Exact test for categorical and Kruskal-Wallis test for continuous data. Univariate analysis followed by multivariate analyses (MVA) with Cox regression analysis including only factors with P-value <0.1 in addition to crucial risk factors were performed to identify independent predictors of toxicity endpoints whenever feasible. A two-sided P-value of <0.05 was considered statistically significant. All statistical analyses were performed using Statistical Analysis Software, version 9.4 (SAS Institute, Inc. Cary, NC, USA).

## Results

We identified 257 patients who met our inclusion criteria after excluding those who received hypo-fractionated course, those treated with a brachytherapy boost, in addition to cases with inadequate follow-up. Arm-A encompassed 72 cases (28%) while Arm-B patients formed the rest (n=185; 72%). For the entire cohort, median age was 73 years (48-85), African-Americans formed 53.3% and CCI of 2+ was detected in 49%. With a median PSA of 8.8 ng/dl and T2b-T3b of 15.1% and a median total Gleason score of 7 (6-10); NCCN intermediate-risk constituted 66.9% (n= 172) of the study candidates.

Arm-A received IMRT delivered via VMAT with a median of 2 arcs and 1 arc in phase-1 and II respectively with a median beam time of 2 minutes. Arm-B cases IMRT was underwent with step-and-shoot technique with a median of 9 fields/phase (6-11) delivered in a median of 5.5 minutes. ADT was utilized by 46.7% of all patients with a mean total duration of 8 months (2-40). 

Study arms were well-balanced for most of the baseline characteristics as well as treatment details except that Arm-A included less Grade Group 4/5 and more Grade Group 3 cases (P=0.014) as demonstrated in Table.1. Besides, Arm-A received significantly higher median total dose (79.2 vs 74 Gy), were treated more with 1.8Gy/fraction and more commonly had pelvic lymph-nodes irradiated; P<0.001 for all. A trend for getting close (borderline) to or beyond predetermined constraints for both bladder (P=0.07) and rectum (P=0.08) was detected higher in Arm-A as depicted in Table.2 which may be due to higher delivered total dose. 

**Figure 1:**
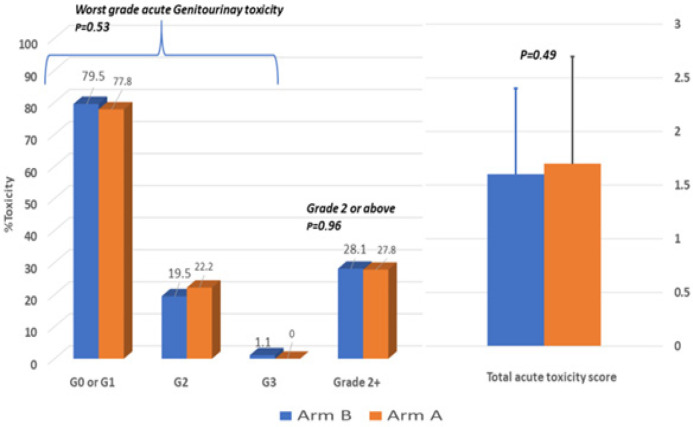
Acute Radiotherapy-Induced Toxicity between Arm-A (VMAT-IMRT with daily CBCT; N=72) and Arm-B (static IMRT without daily CBCT; N=185) measured during weekly visits during radiotherapy up to three months post-therapy with worst grade recorded per RTOG/CTCAE

A great proportion of the study cohort suffered from acute RT-induced side-effects with 54 cases developing G-2 GU toxicities (21%) and only 2 cases (0.7%) having G-3, whereas, only 21 patients had G-2 acute GI toxicity (8.2%) with no G-3 detected. Albeit, acute toxicity did not differ significantly across treatment arms as shown in Figure.1 including the total acute toxicity score formed of worst acute GU + GI +Skin toxicities. 

**Figure 2:**
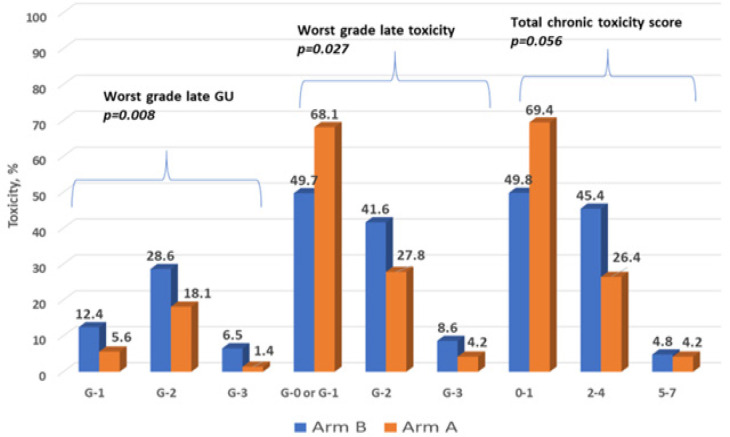
Late Radiotherapy-Induced Side-Effects between Arm-A (VMAT-IMRT with daily CBCT; N=72) and Arm-B (static IMRT without daily CBCT; N=185) measured during radiotherapy and urology surveillance visits after three months post-therapy up to latest follow-up with worst grade recorded per RTOG/CTCAE

Late GU toxicities occurred in 25.7% (n=66) and 5.1% (n=13) for G-2 and G-3 respectively. The most common late G2 GU toxicities were irritative symptoms (n=26; 39.4%), hematuria (n=23; 34.8%) and urethral stricture (n=19; 28.8%); with another six G-3 cases requiring hospitalization and intervention for hematuria and/or stricture. Arm-A had significantly less G-2 (18.1% vs. 28.6) and G-3 (1.4% vs. 6.5%) late GU toxicity than Arm-B (P=0.008) as portrayed in Figure.2. Erectile dysfunction of G2 or more was encountered in 50 patients in the whole cohort (19.5%), albeit rates did not differ between the study arms (P=0.39).

Regarding late GI side-effects; G-2 and G-3 were observed in 19 (7.4%) and three (1.2%) subjects respectively. The prevalent late G-2 GI toxicity was RT proctitis presenting with frequent rectal bleeding in 10 cases (G2/3) of whom 2 underwent endoscopic laser ablation (G-3). Nonetheless, late GI side-effects were similar between prospective vs. retrospective arms (8.4% vs. 8.6; P=0.10).

Over and above, worst grade late RT-related toxicity and the total late toxicity score (worst GU + GI + erectile dysfunction) were both worse in Arm-B (P=0.008 and P=0.056; respectively). On MVA, lack of daily CBCT in Arm-B was independently associated with worst late GU toxicity (P=0.036) after adjusting for RT total dose and pelvic LN irradiation, in addition to CCI group (P=0.04). Also study arm was prognostic for higher total late toxicity score (P=0.002) after adjusting for other factors as shown in Table.3. Meanwhile, African-American race, RT total dose were detrimental for both worst grade late toxicity and total late toxicity score, whereas, higher CCI score was prognostic for higher late toxicity score (P<0.05 for all). Increased RT dose has a protective effect as higher doses were delivered by IGRT using VMAT.

**Table 1:** Baseline Demographic, Clinic-Pathological and Prognostic Characteristics for Prostate-Cancer Patients Receiving IMRT with (Arm-A) or without Daily Image-Guidance (Arm-B)

Characteristic	Arm-A	Arm-B	P-value
	(N= 72; 28%)	(N= 185; 72%)	
Median follow up (years)	3.7 (range 3.3-4.1)	11.2 (range 7-14.6)	<0.001
Median age (years)	71 (range 50-85)	73 (range 48-85)	0.7
African-American race	34 (47.2%)	103 (55.7%)	0.22
Ever smoker (current/ex-smoker)	45 (62.5%)	120 (64.9%)	0.72
Alcohol use			0.41
Social	17 (23.6%)	58 (31.4%)	
Frequent/abuse	11 (15.3%)	30 (16.2%)	
Median baseline CCI score	2 (0-9)	1 (0-7)	0.84
Median AUA /IPSS score at diagnosis	5.0 (2.0-9.0)	5.0 (2.0-10.0)	0.14
PSA level at diagnosis (ng/dl)	11.1 ± 8.9	12.2 ± 10.3	0.39
Grade Group			0.014
1 (3+3) or 2 (3+4)	39 (54.2%)	109 (58.9%)	
3 (4+3)	18 (25%)	29 (15.7%)	
4 (4+4, 3+5 & 5+3) or 5 (4+5, 5+4 & 5+5)	15 (20.8%)	47 (25.4%)	
Median number of positive cores	4.0 (range 1.0-13.0)	5.0 (range 1.0-18.0)	0.14
Mean percentage of positive cores	42.0 ± 25.5	66.6 ± 26.4	<0.001
Clinical T-stage			0.48
T1b-T2c	69 (95.8%)	182 (98.4%)	
T3a-b	3 (4.2%)	3 (1.6%)	
NCCN risk group			0.41
Intermediate	51 (70.8%)	121 (65.4%)	
High	21 (29.2%)	64 (34.6%)	
Median UCSF-CAPRA score at diagnosis	5.0 (2.0-9.0)	5.0 (2.0-10.0)	0.14

IMRT, Intensity-Modulated Radiation Treatment; CCI, Charlson Comorbidity-Index; IPSS/AUA, International Prostate Symptom Score / American Urological Association; PSA, Prostatic Specific Antigen; NCCN, National Comprehensive Cancer Network; UCSF-CAPRA, University of California, San Francisco Cancer of the Prostate Risk Assessment

**Table 2:** Radiotherapy and Androgen-Deprivation Therapy Details for Prostate-Cancer Patients Treated with IMRT with (Arm-A) or without Daily Image-Guidance (Arm-B)

Characteristic	Arm-A (N= 72)	Arm-B (N= 185)	P-value
Median total radiotherapy dose (Gy)	79.2 (78.0-80.0)	74.0 (70.0-78.0)	<0.001
Dose per fraction (Gy)			<0.001
1.8	53 (73.6%)	25 (13.5%)	
2	19 (26.4%)	160 (86.5%)	
Pelvic lymph-node irradiation	13 (18.1%)	1 (0.5%)	<0.001
Rectum dose/volume within constraints			0.08
No	3 (4.3%)	9 (4.9%)	
Borderline	17 (24.3%)	13 (7%)	
Bladder dose/volume within constraints			0.067
No	9 (12.9%)	4 (2.2%)	
Borderline	4 (5.7%)	27 (14.6%)	
Mean PTV maximum dose (%)	105.4 ± 3.1	104.5 ± 2.7	0.12
ADT administration	37 (51.4%)	83 (44.9%)	0.35
Mean ADT total duration (months)	12.1 ± 7.2	11.1 ± 9.1	0.58
Mean months between ADT and RT	2.6 ± 4.2	3.5 ± 2.4	0.15

IMRT, Intensity-Modulated Radiation Treatment; PTV, Planning Target Volume; ADT, Androgen Deprivation Therapy; RT, Radiotherapy

**Table 3:** Multivariable Cox Regression Analysis Models for Predictors of Late Radiotherapy Induced Toxicity for the Whole Study Cohort [n=257]

Variable	Response	Worst late GU toxicity		Worst grade late toxicity^a^		High total late toxicity score^b^	
----	----	OR (95% CI)	P-value	OR (95% CI)	P-value	OR (95% CI)	P-value
Study arm	Arm-B vs. Arm-A	0.42 (0.18-0.92)	0.036	1.65 (0.54-5.22)	0.384	0.67 (0.52-0.87)	0.002
Race	Caucasian vs AA	-------	------	1.87 (1.06-3.33)	0.033	1.78 (1.07-3.08)	0.031
RT total dose^c^	Continuous	0.89 (0.77-1.03)	0.13	0.84 (0.71-0.98)	0.029	0.52 (0.38-0.71)	<0.001
CCI group at diagnosis	CCI-0 vs. CCI-1	1.82 (0.82-4.05)	0.14	1.26 (0.56-2.84)	0.581	1.97 (1.00-3.95)	0.051
	CCI-0 vs. CCI-2+	1.98 (1.03-3.93)	0.044	1.59 (0.70-3.62)	0.267	1.69 (0.88-3.35)	0.121

GU, Genitourinary; OR, Odds Ratio; CI, Confidence Interval; AA, African-American; RT, Radiotherapy; CCI, Charlson Comorbidity Index; ^a^, Adjusted for NCCN risk category and Pelvic lymph-node irradiation; ^b^, Adjusted for clinical T-stage, Pelvic lymph-node irradiation, PTV-maximum dose.

## Discussion

In our study we were able to highlight safe delivery of an escalated curative dose to a homogenous group of intermediate and high-risk localized prostate cancer using IMRT with daily IGRT via CBCT with much improved late toxicity profile and equivalent acute side-effects despite similar dosimetry, when compared to a retrospective similar group who had received IMRT with irregular image guidance. This effect was further proven in MVA after adjusting for other factors.

Our findings are in accordance with Zelefsky et al., (2012) who compared IMRT with or without daily position correction with kV imaging for fiducials and reported more intense late GU toxicities with similar other early and late toxicities. Rates of G2+ late GU side effects in our work were 19.5%vs. 35.1% (P=0.008), for IGRT vs. no IGRT; were less than those in Zelefsky et al., (2008)’s study (10.4% vs 20%; P=0.02), albeit a much higher dose was delivered (86.4 Gy). Lack of daily IGRT and IPSS predicted worse late GU toxicity in MVA similar to our work, nonetheless IPSS was not consistently reported for all candidates (20%) of our study and could not be incorporated to the current MVA. Chung et al., (2008) showed that daily kV imaging of fiducial markers was associated with lower acute GU and GI toxicity in contrast to our work. Nevertheless, all patients of this trial received pelvic RT with a prostatic boost and PTV margins were lower in the IGRT arm (3mm vs. 1cm circumferentially), unlike the current study in which PTV margin was same in both arms (1cm all around and 0.5 posteriorly). It is noteworthy to mention that having equivalent acute toxicity within our study arms implies improved acute toxicity bearing in mind the significantly higher total dose in the daily CBCT arm (79.2 vs. 74 Gy; P<0.001). Like ours, Becker-Schiebe et al., (2016) compared higher to a lower dose (77.4 Gy vs. 72 Gy) with or without IGRT. They depicted significantly better acute and similar late RT-related toxicities albeit higher total dose. However, only 16% received IMRT in contrast to the current work with IMRT in the whole cohort. 

Taking in consideration the extra cost for daily IGRT, two European randomized trials compared daily versus weekly IGRT for setup correction (de Crevoisier et al., 2018; Tondel et al., 2018). This extra cost for daily image online guidance includes the price of technology acquisition, more staff work time for each case, more quality assurance and maintenance time, and also less number of patients per machine due to increased treatment time (Perrier et al., 2013). In the French trial which randomized 470 patients to weekly versus daily IGRT using CBCT (77%), fiducials (EPID or kV) or US to deliver a median of 78Gy, only acute and late GI toxicities were improved (P=0.027), whereas GU toxicities were non-different. Nonetheless, the French control arm had same total dose (78Gy) and IMRT with step-and-shoot technique was utilized in 2 thirds of the cases with 30% receiving 3D-CRT, unlike ours with VMAT for IMRT delivery in all IGRT cases and with a different median dose of 79.2 vs. 74 Gy in the control group. Also our work included LN irradiation in 18% of the IGRT group with potentially more GI toxicities vs only one case (0.5%) in the other arm, in contrast to prostate only RT in the French trial (de Crevoisier et al., 2018). Furthermore, the other randomized phase 3 study (RIC-trial) randomized 257 patients to receive 78 Gy with either daily CBCT or weekly orthogonal portal images. Like our work, no differences were portrayed for acute toxicities even though PTV margins were much less in the daily CBCT arm (7mm all around vs 15 mm). It is important to note that the RIC-trial toxicities were derived using patient reported outcomes after RT in comparison to baseline symptoms rather than physician assessed side effects used in this work. Higher acute hematuria (P=0.02) and nocturia (P=0.04) with weekly IGRT did not reach the pre-specified significance level of P=0.01 (Tondel et al., 2018). Zhong et al., (2014) compared IGRT using CBCT (2-3 times/week) with a retrospective arm treated using IMRT without IGRT and yielded similar acute and late side effects for both arms. This reinforces that our control arm which included infrequent IGRT (weekly or every 2-3 days) is not better than IMRT without IGRT.

Another aspect in this study is that all IGRT cases received IMRT exclusively via VMAT with 2 arcs for most of the treatment time unlike the control arm that received step-and-shoot technique. In fact, this difference may have an influence on our outcomes. A dosimetric Korean study compared both modalities used in our study as well as Tomotherapy and Proton. Authors concluded that better PTV coverage and superior rectal and bowel sparing in high dose volumes were attained with VMAT compared to other modalities albeit all of them achieved the desired constraints (Lee et al., 2015). Nevertheless, no dosimetric advantage was reported in this work. Poon et al., (2013), compared VMAT (2 arcs) vs. step-and-shoot and results depicted that VMAT is delivered in a significantly shorter time and less monitor units by 61% and 48% respectively in addition to better PTV homogeneity index with similar rectal and bladder dosimetry. Another study portrayed mean delivery time of 2 Gy using VMAT (2 arcs) within 2.78 minutes compared to 4.8 minutes for step-and shoot (7-fileds) (Sze et al., 2012). Hence, shorter beam time in VMAT (2 minutes), vs. Step-and-Shoot (5.5 minutes) may have contributed to our findings owing to potentially less intrafractional movement with the shorter VMAT.

Daily image guidance with potential correction of position according to the visualization of PTV soft tissue will take care of setup error and inter-fraction displacement. However, intra-fractional motion still needs to be addressed. Mayyas et al., (2014) compared four different modalities for IGRT (CBCT, US, kV planner images and electromagnetic transponder, Calypso) for daily localization in a group of 27 patients. Intra-fractional motion was assessed using Calypso in 19 cases. The study proposed a minimum margin of 6.6 mm (anterior/posterior), 6.8 mm (superior/inferior) and 3.9 mm (left /right) to compensate for inter-fraction error (4 mm) as well as intrafracion motion if IGRT is used with no preferred modality. Thus, image guidance coupled with VMAT with short RT delivery time will deem the intra-fractional prostatic motion, changes in bladder filling and rectal surface to be with better control.

Other than the established role of daily IGRT, late RT induced toxicities were independently influenced by African-American race as well as by increased comorbidity assessed by CCI per our exploratory MVA model. Higher baseline CCI score of 2+ prevailed in almost half of our total cohort emphasizing the extra attention needed to manage RT side-effects in these patients. Hamstra et al., (2013) showed that late RT toxicity was significantly correlated with comorbidity. Nevertheless, only 27% received IMRT vs 100% in ours. Furthermore, a Canadian population-based cohort demonstrated an independent role of comorbidity in the development of late side-effects indicating hospital admissions, urological or anorectal procedures in accordance with our findings (Nam et al., 2014).

While we present one of the largest cohorts for a single institution with only intermediate and high risk PCa treated with IMRT, some limitations of this study should be listed. First, comparing with a retrospective group is associated with selection and reporting bias. Second, the prospective arm had a relatively short follow-up with possible more intense late RT-related toxicities down the road. Also, we might have seen more dramatic difference between the study arms if the same RT total dose was utilized which was not possible taking into consideration the established role for dose-escalation nowadays with recommended doses of around 80 Gy; which was not the case between 2005-2007. Again, this was due to the establishment of daily IGRT 2008 onwards in our institute, that prevented a more recent comparative arm. Albeit, we had more detailed treatment and toxicity data much better than commonly used databases like SEER and the NCDB. Finally, we relied only on physicians’ notes for grading of RT-induced adverse events with lack of patient filled quality of forms which were not available for the entire cohort. These forms such as Expanded Prostate Index Composite questionnaire (EPIC) reflect the patient’s tolerability, bother level and impact of toxicity on daily functioning and are being increasingly adopted and incorporated within endpoints of many published prospective randomized phase 3 trials (Wortel et al., 2016; Brunner et al., 2019).

In conclusion, for patients with localized prostate cancer of intermediate or high-risk, our study suggests that daily image guidance needs to be an essential component in the process of IMRT delivery of contemporary escalated RT doses due to significantly improved late toxicity profile especially for the late genitourinary side-effects with no impact on acute toxicity. African-Americans and those with higher baseline comorbidities should receive more meticulous care during the radiotherapy course and all along follow up as they have relatively higher tendency to develop significant late toxicities.
